# Therapeutic Agent Delivery Across the Blood–Brain Barrier Using Focused Ultrasound

**DOI:** 10.1146/annurev-bioeng-062117-121238

**Published:** 2021-03-22

**Authors:** Dallan McMahon, Meaghan A. O’Reilly, Kullervo Hynynen

**Affiliations:** 1 Physical Sciences Platform, Sunnybrook Research Institute, Toronto, Ontario M4N 3M5, Canada; 2 Department of Medical Biophysics, University of Toronto, Toronto, Ontario M4N 3M5, Canada; 3 Institute of Biomaterials and Biomedical Engineering, University of Toronto, Toronto, Ontario M4N 3M5, Canada

**Keywords:** ultrasound, brain, therapy, drug delivery, blood–brain barrier, image-guided therapy

## Abstract

Specialized features of vasculature in the central nervous system greatly limit therapeutic treatment options for many neuropathologies. Focused ultrasound, in combination with circulating microbubbles, can be used to transiently and noninvasively increase cerebrovascular permeability with a high level of spatial precision. For minutes to hours following sonication, drugs can be administered systemically to extravasate in the targeted brain regions and exert a therapeutic effect, after which permeability returns to baseline levels. With the wide range of therapeutic agents that can be delivered using this approach and the growing clinical need, focused ultrasound and microbubble (FUS+MB) exposure in the brain has entered human testing to assess safety. This review outlines the use of FUS+MB-mediated cerebrovascular permeability enhancement as a drug delivery technique, details several technical and biological considerations of this approach, summarizes results from the clinical trials conducted to date, and discusses the future direction of the field.

## INTRODUCTION

Despite substantial investigative attention, the global burden of neurological disorders continues to rise (1). These pathologies have immense impacts on afflicted individuals and their families and consume considerable economic resources to deal with symptom management, patient care, and palliation (1). While mechanistic understanding of dementias, brain malignancies, and other neuropathologies continues to expand, the development and implementation of effective treatment strategies have lagged behind. For many disorders, this slow progress may be due to complex or heterogeneous etiologies and/or a lack of viable treatment targets; however, beyond these difficulties remains the challenge of achieving targeted delivery of therapeutic agents to the brain (2).

### Structure and Function of the Blood–Brain Barrier

Specialized vasculature of the central nervous system (CNS) limits more than 98% of small molecule drugs and nearly all large molecule drugs from reaching the brain parenchyma in therapeutically relevant concentrations from systemic circulation (3). A collection of features in the brain and spinal cord, termed the blood–brain barrier (BBB) and blood–spinal cord barrier (BSCB), respectively, play major roles in establishing this selective permeability (reviewed in 4). These features act to maintain the stable and narrow conditions in the CNS that are ideal for neural function.

**Figure 1:**
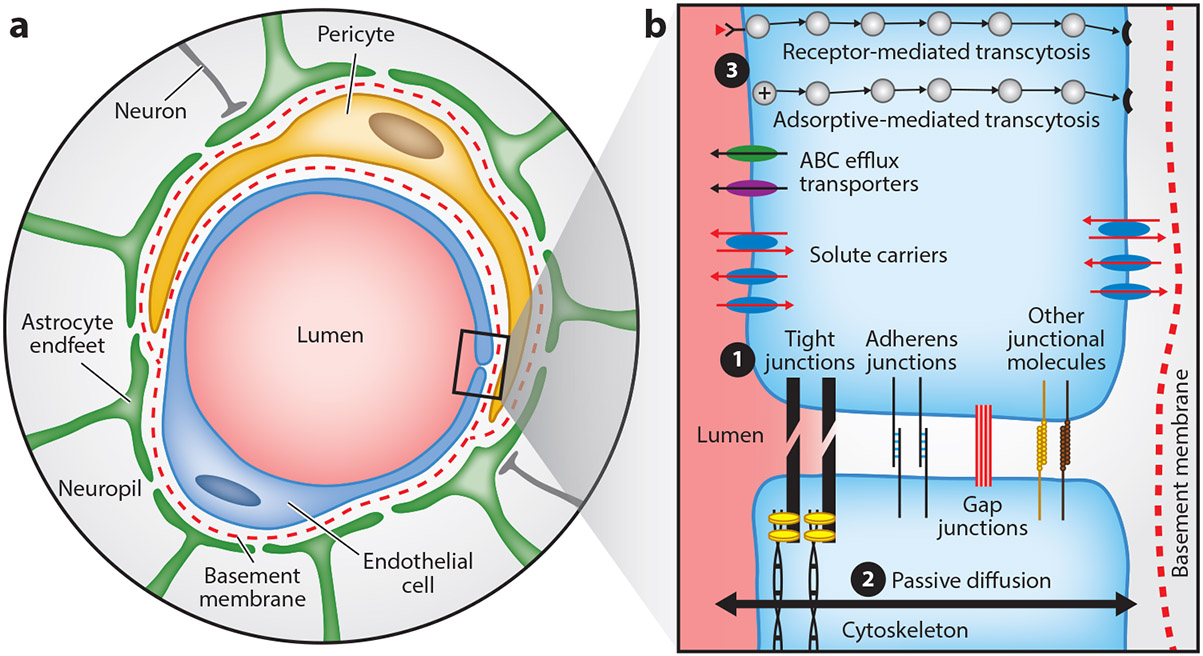
Key components of the BBB. Components of the BBB allow fine control over the substances that transit from systemic circulation to brain parenchyma and vice versa. (*a*) Endothelial cells are surrounded by two basement membranes, an endothelial and a parenchymal basement membrane, composed mainly of collagen IV, laminin, nidogen, and perlecan; pericytes reside in between these layers. Astrocytic endfeet surround the parenchymal basement membrane. Neurons communicate bidirectionally with various cells of the neurovascular unit, primarily astrocytes. (*b*) Specialized endothelial cells (*blue*) line cerebral vasculature, linked together by (❶) tight junction proteins (e.g., claudin-5 and occludin), adherens junction proteins (e.g., vascular endothelial cadherin and platelet endothelial cell adhesion molecule-1), gap junctions (e.g., connexin-30), and other junctional molecules (e.g., endothelial cell adhesion molecule and junctional adhesion molecule-A). Routes of transcellular transport across endothelial cells include (❷) passive diffusion (generally, molecular weight <500 Da and log P_OCT_ = ~2‒4), (❸) receptor-mediated transcytosis (e.g., transferrin receptor-mediated), adsorptive-mediated transcytosis (e.g., histone and tat-derived peptides), solute carriers (e.g., glucose via glucose transporter-1), and ABC-family efflux transporters (e.g., paclitaxel via p-glycoprotein). Abbreviations: ABC, ATP-binding cassette; ATP, adenosine triphosphate; BBB, blood–brain barrier. Figure created based on information detailed by Sweeney et al. (4).

In the brain, specialized, nonfenestrated, endothelial cells (ECs) line blood vessels, linked together by adherens junctions (e.g., vascular endothelial cadherin and platelet endothelial cell adhesion molecule-1), gap junctions (e.g., connexin-37), scaffolding proteins (e.g., zonula occludens-1), and tight junctions (e.g., occludin and claudin-5). These transmembrane and anchoring proteins enable strong bonds between cells, which limit paracellular diffusion (i.e., between cells) and allow for intracellular communication (4). The abluminal sides of ECs are surrounded by basal laminae and, at the level of capillaries, astrocytic endfeet and pericytes (Figure 1). These features contribute to the physical barrier between systemic circulation and brain parenchyma but are also critically involved in controlling local perfusion and maintaining barrier properties (5).

Cerebrovascular ECs also contribute to selective permeability by way of reduced endocytosis and the diverse expression of efflux pumps (e.g., p-glycoprotein) and enzymes (e.g., cytochrome P450). These features act to restrict, regulate, and break down substances that are able to diffuse through cells (i.e., transcellular diffusion), beyond the physical restrictions imposed by the EC layer (i.e., molecular weight <500 Da; log P_OCT_ = ~2–4) (6). The BBB also participates in the coordination of immune responses in the brain by providing a niche for the chemoattraction of circulating granulocytes and peripheral blood mononuclear cells (7); however, under normal physiological conditions, the brain exhibits low levels of immune cell infiltration relative to other organs (7). Together, the specialized features that comprise the BBB act to preserve efficient function and protect from infection. While key differences exist, many of these features are also present in the BSCB, contributing to similar difficulties for drug delivery to the spinal cord.

### Current Methods of Drug Delivery to the Brain

The challenge of delivering drugs to the CNS has motivated the development of a variety of strategies to overcome this problem, each with attributes and drawbacks. Generally, these strategies either aim to bypass the barrier features of the cerebrovasculature or co-opt endogenous mechanisms. Perhaps the simplest approach, direct intracranial injection, has been shown to produce therapeutically relevant drug concentrations with a high degree of spatial precision (8); however, due to limited diffusion from the site of injection and the risks associated with penetrating injury (9), direct intracranial injection approaches are not well suited for the treatment of large tissue volumes [i.e., greater than a cubic centimeter (10)] or conducive to repeated administrations. Conversely, both intracerebroventricular and intranasal administration result in relatively low concentrations and heterogeneous distributions within the brain, owing to a reliance on bulk flow and diffusion within cerebrospinal fluid and interstitial fluid (11, 12).

Alternatively, chemical stimuli have been employed to modify cerebrovascular permeability by promoting paracellular diffusion, thus providing a route for systemically administered drugs to enter the brain. Clinical trials have demonstrated enhanced delivery of chemotherapeutic agents in patients with metastatic brain cancers via intracarotid infusions of hyperosmotic solutions, such as mannitol (13). While this approach may be well suited for the treatment of diffuse brain cancers, a lack of fine targeting and significant risks, including vascular damage (14), neuropathological changes (13), and seizures (13,15), may limit its flexibility as a general drug delivery strategy for the brain.

Beyond physically bypassing or increasing the permeability of cerebrovasculature, novel drug development and modifications to existing therapeutics have been employed to increase drug delivery to the brain. The development of small (<400 Da), lipid-soluble therapeutic agents with low affinities for efflux transports and low systemic toxicity are ideal for BBB penetration; however, balancing these physical features while maintaining pharmacological action is not always feasible (2). To enhance BBB permeability of existing therapeutics, several modification strategies have been employed, including increasing lipid solubility (2) or leveraging endogenous receptor-mediated transport systems (16). While these approaches may prove beneficial for specific applications, the maintenance of therapeutic action after drug modifications can pose a challenge. Additionally, achieving spatial specificity within the CNS without the use of additional biological or physical targeting techniques is difficult.

A variety of other strategies for increasing therapeutic agent delivery to the brain have been tested (17-19), each with advantages and limitations. Ultimately, the development of flexible strategies to overcome the challenges posed by the BBB and BSCB, while maintaining a low risk of inducing damage, remains a challenge. Compounding this problem is the difficulty of engineering an approach that can aid in the treatment of both highly localized pathologies, by producing localized drug delivery, and those with more diffuse distributions, by producing more widespread delivery. Given the variety of neuropathologies for which drug delivery currently presents an impediment to treatment and the number of individuals impacted by these afflictions, the need for more rapid progress is high. This review explores the use of focused ultrasound and microbubble (FUS+MB)-mediated vascular permeability enhancement as a promising approach that seeks to address these challenges.

## FOCUSING THROUGH BONE

The use of focused ultrasound for neurosurgical interventions dates to the 1950s when the Fry brothers were performing clinical studies using high-intensity exposures (20); however, the need for invasive craniotomy greatly hindered the adoption of ultrasound brain therapy. Bone is highly attenuating and distorting to ultrasound due to a strong impedance mismatch (i.e., much greater density-dependent speed of sound than in the adjacent soft tissues) as well as heterogeneity associated with bone density, structure, and thickness (21, 22). Consequently, focusing through the skull is challenging. While some applications of therapeutic ultrasound might leverage existing bone windows, as is the case following resection surgery (23), broad clinical adoption of this technology, and particularly for diffuse or widespread pathology, relies on transcranial methods that leave the bone intact.

Several technological advances have enabled noninvasive brain therapy through intact bone. First, lower ultrasound frequencies better penetrate the skull, as they undergo lower attenuation and, due to the longer wavelengths, smaller phase shifts (24). Consequently, most clinical-scale work focuses on submegahertz frequencies. The development of low-frequency, large, multielement phased arrays allowed for electronic correction of the beam distortions by individually adjusting the phase of the acoustic wave generated by each element such that they arrive in phase at the focus (25). Second, these phased arrays, in combination with the methods to calculate required phase and amplitude corrections from patient-specific X-ray computed tomography data (24, 26), have enabled precise transcranial focusing. Online magnetic resonance imaging (MRI)-based targeting and thermometry are now in routine clinical use for thermal ablation of essential tremor in patients, demonstrating the feasibility of this approach for achieving transcranial ultrasound focusing (27, 28). Similarly, phase corrections have been achieved in lab experiments through the use of rapid prototyping to 3D-print subject-specific acoustic lenses to be used in conjunction with inexpensive single-element transducers (29). This approach is lower in cost but sacrifices the flexibility offered by phased array devices.

## SONICATION AND MICROBUBBLE PARAMETERS AFFECTING CEREBROVASCULAR PERMEABILITY

Preformed gas microbubbles, with diameters on the order of micrometers, have for decades been used as ultrasound contrast agents in diagnostic imaging to assess blood flow. These microbubbles compress and expand during the high and low phases of a pressure wave, respectively, due to the high compressibility of the encapsulated gas relative to surrounding fluid. This contraction and expansion happens at the frequency of the ultrasound wave (i.e., 200–500 kHz for clinical treatments) and causes fluid streaming around each microbubble (30), which in turn is associated with shear and circumferential stresses being exerted on blood vessel walls. With an increase in ultrasound pressure amplitude, microbubble oscillations become nonlinear, generating acoustic emissions at harmonics of the driving frequency. At higher pressure amplitudes, unstable oscillations lead to subharmonic (i.e., 1/2 of the driving frequency) and ultraharmonic (i.e., 3/2, 5/2, etc. of the driving frequency) emissions. A further increase in the pressure amplitude will result in violent microbubble collapse with associated wideband frequency emissions and mechanical and thermal stresses (31). This collapse, termed inertial cavitation, can result in tissue damage (32). Microbubble oscillations, and thus the induced vascular effects, depend on the physical properties of the microbubble and the blood vessel walls as well as the acoustic parameters of the ultrasound exposure.

A wide range of exposure parameters for enhancing cerebrovascular permeability with FUS+MBs have been explored. Early studies examined the effects of parameters such as frequency, pressure, burst length, burst repetition frequency, total sonication duration, and microbubble type, size distribution, and dose (27, 28, 33-38). Although there are discrepancies in the literature, some general claims can be made. First, the threshold for enhancing BBB permeability varies with frequency, with lower frequencies requiring lower peak negative pressures (PNPs) to produce an observable effect. McDannold et al. (34) found that the mechanical index, the ratio of PNP to the square root of frequency (39), could serve as a unifying parameter to relate results across frequencies. Second, while longer bursts can produce greater increases in permeability (35), there is a limit beyond which increases in burst length no longer yield greater permeability enhancement (33), likely due to the depletion of the microbubbles during the burst. Third, burst repetition frequency must also be carefully selected, with consideration of the burst length being used, to allow for adequate replenishment of microbubbles in the focal volume between bursts (38).

The discrepancies in the literature regarding the effects of specific parameters can, in part, be attributed to a high degree of variability in preclinical studies, resulting in some trends being described as statistically significant in some reports but lacking significance in others. With regard to discrepancies in specific values, such as the PNP threshold required to induce a change in cerebrovascular permeability, there are a multitude of factors to be considered, including but not limited to transducer calibrations and substantial uncertainty in pressure amplitude in the closed rodent skull cavity where standing waves can form at low frequencies (40, 41). This last point explains why the pressure amplitude required to generate cerebrovascular leakage in large animal models with portions of the skull removed is about twice what is required in small animals with closed skulls. In general, the field should shift away from using fixed-pressure exposures to those where pressure is actively modulated to elicit specific microbubble behavior; these approaches are discussed in a subsequent section.

Equally as important as the choice of ultrasound parameters is the appropriate selection of microbubble dose and proper handling. Early work in the field established that microbubble dose can impact the amount of tracer delivered to brain tissue (28) and that microbubble size can also impact permeability (36, 42). Given the difficulty in comparing studies using different microbubble populations, Song et al. (43) have recently proposed microbubble gas volume as a unifying parameter to reconcile results from different studies. They demonstrated, using two distinct monodisperse microbubble populations, that by plotting tracer accumulation as a function of microbubble gas volume instead of microbubble number, they could fit a single curve through both datasets. The situation is, however, complicated by the nonlinear dependency of microbubble oscillations on microbubble size and driving frequency (44).

Further, when isoflurane is used as an anesthetic, the choice of carrier gas has a substantial impact on the resulting permeabilization of the BBB. McDannold et al. (45) demonstrated that the use of oxygen as a carrier gas greatly diminished the leakage of cerebrovasculature and also the strength of recorded microbubble emissions, compared with the use of medical air. These results suggest that the use of oxygen as a carrier gas more rapidly depletes the microbubbles in circulation, consistent with reports from diagnostic ultrasound (46). Finally, it is important to be cognizant of microbubble circulation time relative to the total sonication time. Since a bolus injection will rapidly decay, leaving far fewer microbubbles later in the sonication, there has been a more recent shift in studies toward using longer infusions to produce a more consistent concentration of microbubbles in circulation, particularly in studies looking at active control where consistency of the microbubble emissions over the duration of the sonication is essential (47-49).

When thoughtfully selected, sonication and microbubble parameters can result in reversible modulation of the BBB permeability without evidence of overt tissue damage. Conversely, inappropriate selection can lead to a lack of effect or an overexposure of the tissue, with the potential for permanent tissue changes. The following section details the biological effects that have been observed in response to FUS+MB exposure.

## TISSUE EFFECTS

### Histology

From the earliest studies exploring FUS+MB exposures in the brain, histological evaluations of tissue health have been used to gauge treatment risk. Common stains, such as hematoxylin and eosin (47, 50, 51) and Prussian blue (52, 53), have been, and continue to be, valuable tools for refining sonication parameters and assessing new techniques. As such, studies have reported a considerable range of histological observations in the hours and days following FUS+MB exposure. While some have noted low levels of red blood cell (RBC) extravasations with rare occurrences of ischemic neurons in the first 24 h following sonication (48), others have described persistent vascular dilation, glial scars, and metallophagocytic cells after more than 3 months (54). Likewise, macrophage infiltration, as assessed by hematoxylin and eosin (52, 55-57) and CD68 immunodetection (54, 58, 59), has been reported weeks (52, 54) following sonication; however, at lower PNPs, it is possible to achieve BBB permeability enhancement with no or few macrophages evident in targeted tissue (55, 60). These disparities highlight two key concepts for histological assessment of tissue health following intervention. First, FUS+MB parameters greatly influence both the magnitude of BBB permeability enhancement and the incidence and degree of tissue damage incurred. Second, the interval between sonication and euthanasia substantially affects the opportunity for lesion formation and tissue repair.

### Vascular Integrity

Given that, in the context of FUS+MB exposures, changes in vascular permeability are driven by direct and indirect physical interactions with oscillating microbubbles, it is important to consider how vascular integrity is affected by this intervention. Sheikov et al. (61) recorded electron microscopy observations in the hours following sonication and were the first to report increases in the number of vesicles, vacuoles, fenestrations, and transcellular channels in rabbit cerebrovascular ECs. Both transcellular vesicular trafficking (62) and paracellular leakage (63) of intravenously administered tracers, excluded by the BBB under normal physiological conditions, were observed 1–2 h after FUS+MB exposure. Importantly, changes in tight junction integrity, measured by a reduction in the immunoreactivity of occludin, claudin-5, and zonula occludens-1 in the interendothelial clefts, were no longer present at 4 or 24 h after sonication (63). These observations suggested that FUS+MB exposure generates multiple routes of vascular leakage and that the impacts on tight junction integrity are transient.

Analysis of BBB permeability enhancement using in vivo two-photon microscopy has also provided evidence of distinct types of leakage generated by FUS+MB exposure. Fast leakage (also known as focal disruption or microdisruption), evident during or shortly after sonication, has been characterized as a rapid diffusion of dyes into brain parenchyma at distinct points along blood vessels (64-67). This type of leakage has been reported to occur more frequently in smaller blood vessels (i.e., diameter <30 μm) and is speculated to result from a widening of interendothelial clefts or sonoporation (64, 66, 67). Conversely, slow leakage is characterized by a gradual, diffuse accumulation of dye surrounding vessels (64-67). Whether due to a delay in reaching a detection threshold or a delay in manifestation, slow leakage has typically been observed at least 10 min after sonication and appears to affect a wide range of blood vessel sizes (64-67). Lastly, hemorrhagic leakage has been described as a rapid blurring of vessel edges and a loss of fluorescent signal due to RBC extravasation (64); however, this type of leakage appears to be largely avoidable with the use of appropriate exposure conditions, as few regions of RBC extravasation are observed when PNP is adjusted to limit violent microbubble collapse (inertial cavitation) (32).

**Figure 2:**
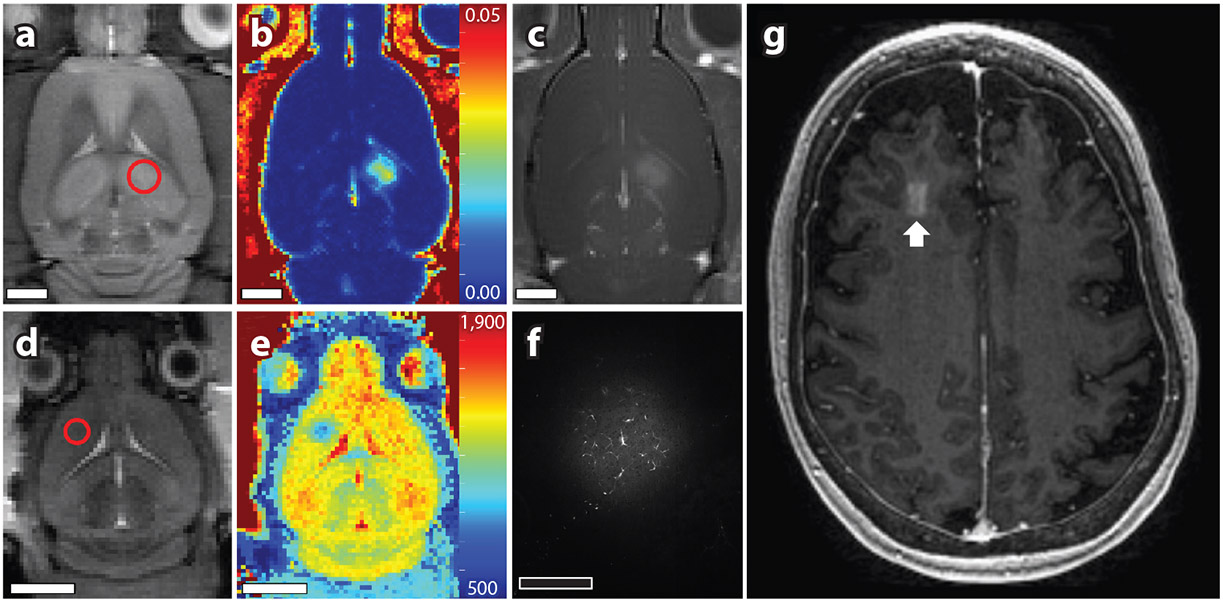
Evaluating FUS+MB-mediated BBB permeability enhancement. A variety of methods have been employed to target desired brain structures and assess cerebrovascular leakage following ultrasound and microbubble exposures. (*a*,*d*) MRI spatial coordinates can be coregistered to a transducer positioning system to allow targets (*red circles*) to be chosen from structural magnetic resonance images (*a*, rat; *d*, mouse). In preclinical studies, BBB permeability enhancement has been quantified postsonication using a number of methods, including (*b*) dynamic contrast–enhanced MRI, (*c*) contrast-enhanced T1-weighted imaging, (*e*) T1 mapping following MRI contrast administration, and (*f*) dextran fluorescence in tissue sections. Images in panels *a–c* and *d–f* were collected from the same animal subjects, respectively. Color bars indicate K^trans^ (min^−1^) of (*b*) gadobutrol and (*e*) T1 in milliseconds. Clinical trials have generally utilized contrast enhanced T1-weighted imaging to confirm changes in BBB permeability. (*g*) A white arrow indicates a region of increased cerebrovascular leakage generated by FUS+MB exposure targeted to the dorsolateral prefrontal cortex in a participant with Alzheimer’s disease. Image in panel *g* adapted from Reference 144. White scale bars: 4 mm. Black scale bar: 400 μm. Abbreviations: BBB, blood–brain barrier; FUS+MB, focused ultrasound and microbubble; MRI, magnetic resonance imaging.

The time course of the aforementioned vascular effects track well with MRI-based analysis of the duration and kinetics of BBB permeability enhancement (Figure 2). Through the use of MR contrast agents of varying sizes, Marty et al. (68) calculated that the time required for the magnitude of BBB permeability to drop by 50% following FUS+MB exposure (i.e., half-closure time) was influenced by hydrodynamic diameter. For contrast agents with diameters of 7 and 1 nm, the half-closure times were found to be approximately 1 and 5 h, respectively. For large therapeutics, such as natural killer cells, extravasation largely occurs during sonication (69). In addition to the size of molecules extravasating, the initial magnitude of BBB permeability enhancement has been shown to influence the duration of this effect (70-72). Thus, depending on exposure conditions and the size of the tracer employed (or therapeutic agent being delivered), the duration of FUS+MB-mediated BBB permeability enhancement can vary greatly, with reports ranging from hours (68, 70) to days (73) of detectable leakage. It is important to note, however, that prolonged increases (e.g., beyond 24 h) in vascular permeability have been associated with substantial tissue damage (73).

In addition to facilitating the movement of substances from systemic circulation into brain parenchyma, FUS+MB exposure may also influence interstitial fluid flow and brain-to-blood diffusion/transport. Through the use of intravital microscopy and mathematical modeling, Arvanitis et al. (74) noted enhanced interstitial drug transport in the brain tumor microenvironment following sonication. This effect may be driven by altered convective transport, resulting in increased penetration of chemotherapeutic drugs into tumor tissue. Others have similarly noted changes in interstitial fluid flow in brain tumors following FUS+MB exposure using MRI-based quantification (75). There is also evidence that FUS+MB exposure may alter the efflux of substances from the brain, as transcription (76) and protein expression (77, 78) of p-glycoprotein have been found to be transiently downregulated for 48–72 h following sonication. Given the contribution of efflux pumps, such as p-glycoprotein, to the poor penetration of many hydrophobic drugs into the brain (79), this effect may contribute to a greater exposure of brain tissue to therapeutic agents delivered with FUS+MBs; however, it is important to note that the movement of substances from brain to blood, via either passive or active mechanisms, may also be increased by this intervention depending on a number of factors, including concentration gradients. Evidence for this effect comes from reports of increased serum concentrations of glial fibrillary acidic acid protein (GFAP), myelin basic protein (80), and reporter gene messenger RNA (81, 82) following sonication. In these studies (80-82), authors have described the potential utility of brain-to-blood transport following FUS+MB exposure for noninvasive characterization of pathological tissue (also known as liquid biopsy).

Together, these studies reinforce the idea that the effects of FUS+MB exposure on BBB integrity and vascular permeability are complex. A wide range of factors relating to both sonication parameters and biological mechanisms influence how cerebrovasculature responds, affecting the kinetics of leakage, distribution of extravasated substances in brain parenchyma, and the return of BBB integrity. Effects can range from a return to baseline permeability within hours of sonication with no lasting indications of vascular damage (70, 83) to persistent vessel leakage evident days following sonication accompanied by substantial RBC extravasations (73).

### Inflammation

Thus far, all data published on the topic have indicated some degree of inflammatory response associated with FUS+MB-mediated BBB permeability enhancement; however, the magnitude, time course, and clinical relevance of these responses have been the subject of much debate, as have the factors that most strongly influence these effects.

One of the earliest descriptions of inflammation following FUS+MB exposure came from Jordão et al. (84), who noted increased ionized calcium-binding adaptor molecule-1 (IBA1) and GFAP immunoreactivity in the hours and days following sonication. These indicators of gliosis were found to dissipate by 4 and 15 days, respectively, in wild-type mice. Conversely, elevated GFAP and IBA1 immunoreactivity has been reported 7 weeks following FUS+MB exposure, accompanied by morphological indications of glial scar formation, when a high-pressure amplitude and microbubble dose are employed (54). This connection between exposure conditions and the magnitude of inflammatory response has been demonstrated for a number of inflammatory indicators, including macrophage infiltration (55, 60) and transcription of proinflammatory cytokines (85).

The first evidence of FUS+MB-induced inflammation at the level of transcription came from microarray analysis of microvessels in the acute stages following sonication. This work reported an upregulation of key inflammatory mediators, such as *Ccl2, C3, Il1b, Il6*, and *Sele*, at 6 h; however, by 24 h, expression levels of these genes largely returned to control levels or were reduced relative to the 6-h time point (76).

In whole-brain tissue, two independent studies (59, 85) have explored the expression of nuclear factor kappa B (NFκB) pathway–related genes following FUS+MB exposure. This pathway is involved in a wide range of processes, including innate and adaptive immunity, inflammation, and stress responses (86). Kovacs et al. (59) first demonstrated a significant upregulation of several drivers of inflammation, including *Il1a, Il1b, Selp, Tnf,* and *Icam1*, in the first 12 h following sonication, accompanied by similar changes in protein expression. This damage-associated molecular pattern was proposed to be a barrier for clinical translation; however, the high microbubble dose used in this work, approximately 10 times that used for clinical imaging, was speculated to influence the magnitude of this response (87). To explore the potential impact of microbubble dose on inflammation, a subsequent study (85) compared NFκB pathway–related gene expression following sonications with a clinical imaging dose and 10 times the clinical imaging dose of microbubbles. When sonicating with the high microbubble dose, changes in gene expression largely matched those reported by Kovacs et al. (59) but were significantly greater than in tissue sonicated with a clinical imaging dose of microbubbles. Importantly, this study (85) also showed that the transcription of several key inflammatory mediators, including *Tnf, Icam1, Ccl5,* and *Il1b*, were correlated to the magnitude of BBB permeability enhancement.

This result is in harmony with a large body of preclinical research demonstrating repeated FUS+MB-mediated BBB permeability enhancement without the generation of overt tissue damage, provided that established sonication parameters are employed. Most notably, McDannold et al. (52) conducted repeated sonications targeted to the visual cortex and the relay system for the visual pathway in rhesus macaques. Following up to five exposures over 5–9 weeks, tests of visual performance, visual acuity, motor skills, and species-specific behaviors revealed no deficits. Similarly, no evidence of histological damage was observed.

While overt tissue damage and behavioral deficits can be avoided, it is important to emphasize that any perturbation of homeostatic conditions in the brain carries with it some degree of risk. The observation of acute inflammation following FUS+MB exposures is somewhat expected, given that the goal of this intervention is to physically promote cerebrovascular permeability. Mechanical stresses on vascular walls and the extravasation of plasma proteins from systemic circulation would be expected to temporarily disrupt homeostatic conditions and contribute to an acute inflammatory response. While the clinical risk of this response may be low, recent work has demonstrated that the administration of dexamethasone, a commonly used glucocorticoid, following sonication leads to a more rapid return of BBB integrity and a significant reduction in the expression of inflammatory mediators (72). Such pharmacological modifications to FUS+MB exposure may prove useful in further widening the safety window of this drug delivery technique.

As with other inflammatory stimuli, if the magnitude or duration of response is sufficient, substantial detriment will result (88). While it is apparent from previous work that the degree of inflammation that follows sonication is related to the magnitude of permeability enhancement (59, 85), other factors may also influence this relationship. Continued investigative effort is needed to better understand factors that drive FUS+MB-induced inflammation, as well as to characterize its impact on long-term tissue health. It is paramount, however, that work in this arena is conducted with parameters that reflect current best practices. Clinical implementation of this approach will require weighing the risks associated with transient inflammation against the benefits expected from therapeutic agent delivery.

### Cell Proliferation

Perhaps causally linked to transient inflammation, distinct trophic processes have also been observed following FUS+MB exposure. Scarcelli et al. (89) first reported increased density of newborn neurons in the dentate gyrus 4 days after unilateral sonication of the hippocampus in adult mice. Subsequent work has found that the increased proliferation and survival of these hippocampal neurons requires generating cerebrovascular leakage, as sonications at low PNP or at high PNP without microbubble administration (i.e., conditions with no detectable effect on BBB permeability) did not produce increases in neurogenesis (90). It has been speculated that FUS+MB-induced neurogenesis, along with increased dendritic branching of granule neurons in the dentate gyrus (91), may contribute to working memory improvements reported following three biweekly sonications in a mouse model of Alzheimer’s disease (AD) (TgCRND8) (91). Similarly, Shin et al. (92) have reported improved spatial memory following FUS+MB exposure in a rat model of cholinergic neuron degeneration (192 IgG-saporin). Behavioral improvements were accompanied by increased hippocampal neurogenesis and elevated mature brain-derived neurotrophic factor and acetylcholinesterase concentrations. These findings may suggest a therapeutic potential of FUS+MB exposure alone for aiding in the repair of damaged neuronal networks.

Beyond neurogenesis, studies have reported between a quarter (92) and half (89) of newborn cells generated following sonication to be non-neuronal. While a portion of these cells were found to be positive for astrocyte markers (89, 92), a substantial proportion have been unidentified in these studies. Of this unclassified population, newborn endothelial cells may make up a small percentage, as indications of a mild elevation in blood vessel density have been reported following unilateral hippocampal FUS+MB exposures (72, 93). These changes were found to peak between 7 and 14 days following sonication, accompanied by increased vascular endothelial growth factor immunoreactivity, and returned to baseline by 21 days (93). Similar effects of FUS+MB exposure on blood vessel density have been observed in skeletal muscle, but of greater magnitude (94, 95). Additionally, the induction of angiogenic processes in the brain following sonication is consistent with bioinformatic analysis of acute gene expression changes in microvessels (76). Taken as a whole, results suggest that FUS+MB-mediated BBB permeability enhancement induces cell proliferation in a number of populations and that these trophic effects may have therapeutic potential.

## THERAPEUTIC APPLICATIONS

### Drug Delivery

The scarcity of drugs able to transit cerebrovasculature from systemic circulation and the growing burden of neuropathologies motivate the development of flexible strategies to address this bottleneck. FUS+MB exposure has been shown to promote the delivery of a large variety of therapeutic agents to targeted regions in the brains of disease models.

One such class of drugs, chemotherapeutics, has been shown to both accumulate in targeted brain tissue following sonication and lead to positive effects in preclinical models. As a proof of concept, Treat et al. (27) first demonstrated the delivery of doxorubicin to the brains of healthy rats. Later, this group reported reduced tumor volumes and increased survival times following FUS+MB-mediated delivery of doxorubicin in a glioma rat model (96). Since these early studies, others have demonstrated similar results with doxorubicin (48, 74, 97-101) as well as enhanced delivery of methotrexate (102), carmustine (103-105), temozolomide (106, 107), carboplatin (108), cytarabine (109), and irinotecan (110). These promising preclinical results motivated the first clinical trials for ultrasound and microbubble–mediated drug delivery (discussed in the section titled Clinical Trials). Given the toxicity of these drugs to healthy brain tissue, targeted delivery is essential to reduce off-target effects.

Another major area that has received much attention in the field has been viral vector-based gene therapy. While BBB-crossing adeno-associated virus (AAV) capsids have been developed in recent years (111), FUS+MB-mediated delivery enables regional specificity of transduction without direct injection (112, 113). Reporter gene expression following FUS+MB-mediated delivery has been demonstrated with AAV9 (53), AAV1 (43), AAV2 (114, 115), AAV1/2 (115), AAV1 and AAV2 (synapsin promoter) (116), and AAV1/2 (GFAP promoter) (113). Beyond these proof-of-concept studies, FUS+MB exposure has been shown to enhance the delivery of an AAV9 vector bearing a short hairpin RNA sequence targeting the α-synuclein gene. Authors observed a significant reduction (>50%) in α-synuclein protein expression in the targeted hippocampus, substantia nigra, and olfactory bulb 1 month following treatment (117). These results suggest that FUS+MB-mediated gene therapy may be an effective tool in the treatment of Parkinson’s disease. Others have demonstrated FUS+MB and AAV-mediated gene delivery for optogenetic (118) and chemogenetic (119) applications. Additionally, nonviral gene therapies have also been combined with FUS+MB exposure via enhanced delivery of small interfering RNAs (120, 121), liposome-encapsulated DNA plasmids (122, 123), and DNA-bearing nanoparticles (124).

Beyond those already discussed, a variety of other therapeutic agents have been delivered to targeted brain regions via FUS+MB exposure. Especially intriguing is the ability to deliver neural stem cells (125) or natural killer cells (69), which may provide a platform for a variety of therapeutic approaches. Other therapeutic agents delivered include antiamyloid beta antibodies (126), antidopamine receptor D4 antibodies (127), Herceptin (128), interleukin-12 (129), erythropoietin (130), and brain-derived neurotrophic factor (131). In addition to characterizing the delivery and efficacy of FUS+MB-mediated therapeutic agent delivery, further work in the field should focus on evaluating the movement of substances that have crossed the BBB. Recent observations of glymphatic clearance of MRI contrast agents following sonication (132) emphasize the dynamic nature of transport within the CNS.

### Non–Drug Delivery Applications

While a large body of research in the field has centered on drug delivery, FUS+MB exposure alone has been shown to generate biological responses that may be therapeutically relevant in specific pathological circumstances. The most studied example comes in the context of AD, where FUS+MB-mediated BBB permeability enhancement has been found to improve proteinopathy and performance in behavioral tests in rodent models. Reductions in amyloid beta (Aβ) plaque size and surface area have been observed 4 days following sonication in TgCRND8 mice, accompanied by increased microglial activation surrounding Aβ plaques as well as greater levels of Aβ within microglia and astrocytes (84). By tracking Aβ plaque size longitudinally with in vivo two-photon microscopy, Poon et al. (133) found that the maximal effect occurs approximately 4 to 7 days following sonication, with plaques returning to baseline size within 3 weeks. With repeated treatments targeted bilaterally to the hippocampus (once per week for 3 weeks), Burgess et al. (91) reported a significant reduction in plaque load, increased proliferation of neural progenitor cells in the dentate gyrus, and improved performance on hippocampal-dependent tasks (TgCRND8 mice). These reports suggest that FUS+MB exposure alone contributes to a transient increase in Aβ clearance, leading to behavioral improvements, and suggest that glial cell phagocytosis may contribute to this effect.

In addition to TgCRND8 mice, other models have also been used to assess the impact of FUS+MB exposure on AD-like pathology. In the K369I tau transgenic K3 mouse model, Pandit et al. (134) observed a FUS+MB-mediated reduction in hyperphosphorylated tau following 15 weekly whole-brain treatments. This effect was found to be driven, in part, by the autophagy pathway in neurons, but not in glia, and was accompanied by improvements in motor function. Similarly, others have observed positive impacts of FUS+MB exposure on AD-like pathology in APP23 (58, 135), pR5 (136), and rTg4510 (137) mouse models. As a whole, research into this effect suggests that FUS+MB exposure may have the potential to transiently slow the accumulation of protein aggregates associated with AD, leading to improved cognitive function. Further research in the field would benefit from continued characterization of the mechanisms driving these effects, optimizing exposure conditions for specific biological responses, and the development of combinatorial treatment strategies to enhance the efficacy of this approach.

## MONITORING AND CONTROL

The wide range of bioeffects that can be elicited with ultrasound in combination with microbubbles, as well as the variability of the ultrasound propagation through human skull, underscores the need for robust monitoring and control of treatment exposures to ensure that the desired reversible modulation of BBB permeability is achieved. McDannold et al. (32) were the first to report an association between recorded microbubble emissions and successful generation of BBB leakage. They observed an increase in harmonic emissions at the second and third harmonics when cerebrovascular permeability enhancement was achieved and also were the first to report that this effect can be achieved without indicators of inertial cavitation (32). Others (138, 139) have also reported harmonic emission changes, as well as associated sub- and ultraharmonic emissions, and/or broadband emissions with histological damage.

Several feedback control algorithms have been developed and tested in animal models to promote consistent, transient increases in BBB permeability without overt tissue damage (47-49, 140-143). Although these controllers vary in the types of emissions that are used to establish effective operating pressure amplitudes, the common goal is to ensure adequate microbubble excitation to elicit a change in BBB permeability while avoiding overexposure of the tissue. Each implementation has shown promising results.

**Figure 3:**
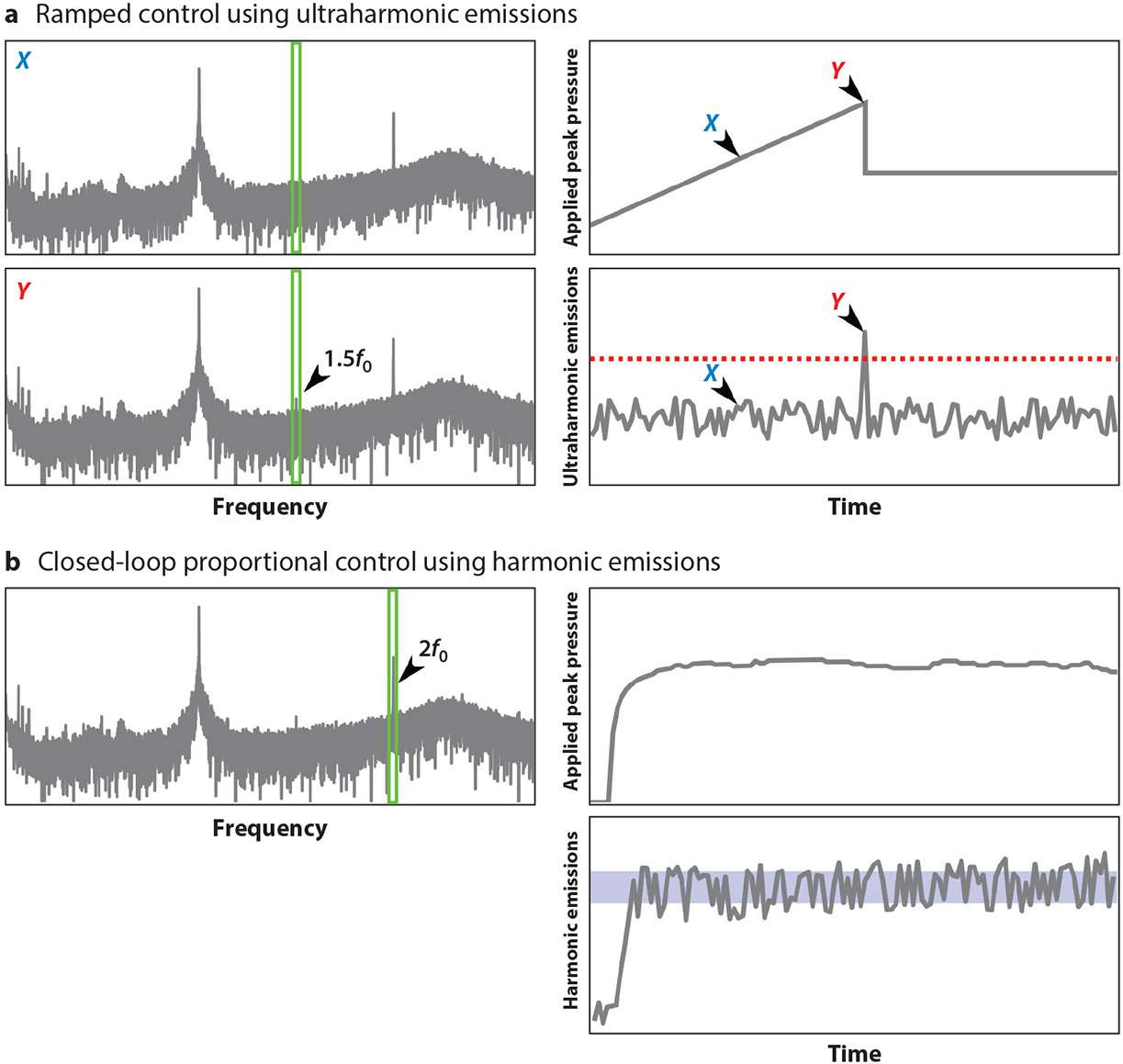
Conceptual illustration of active control strategies. (*a*) The ramped control scheme, based on ultraharmonic emissions, increases the pressure burst to burst until detection of a threshold (*red dashed line*) change in emissions from the integrated band around the ultraharmonic frequencies (*green boxes*, 3/2 *f*_0_). At time *X*, there is an absence of signal in this band, while at time *Y*, the presence of ultraharmonic emissions triggers a decrease in applied pressure to a predetermined target level (percentage of the peak reached) for the remainder of the sonication duration. (*b*) The proportional control scheme, based on harmonic emissions, integrates the harmonic signal over bands around one or multiple harmonics of the driving frequency (illustrated here with a *green box* around the second harmonic, 2*f*_0_). The applied pressure is adjusted from burst to burst to maintain the harmonic emissions within a target zone (*blue band*).

One class of approaches utilizes sub- or ultraharmonics to determine operating pressure ranges. O’Reilly & Hynynen (47) presented the first active feedback control algorithm for BBB permeability enhancement, incrementing the pressure burst to burst until the onset of ultraharmonic emissions, at which point the pressure would be decreased to a target percentage of the peak pressure reached and maintained for the remainder of the sonication (Figure 3). A version of this controller has been successfully implemented in clinical studies (144). In a similar approach, Tsai et al. (141) used the onset of subharmonic emissions as a predictor of BBB leakage; however, they employed fixed pressure sonications and terminated the sonication at the onset of subharmonic emissions. In both of these approaches, sustaining the sub- or ultraharmonic emissions is avoided—the sonication is terminated or pressure decreased immediately—as continued exposure at these levels can induce tissue damage (47). Interestingly, in two recent studies (142, 145), authors have sought to achieve sustained sub- and ultraharmonic emissions with their controllers. These studies lack detailed histology, so it is difficult to ascertain if tissue damage is being induced. However, they also differ from other studies in that they employ nanobubbles rather than microbubbles. Given the large disparity in microbubble size, relationships between recorded emissions and observed bioeffects reported in other studies utilizing microbubbles may not hold for these smaller nanobubbles.

A separate class of approach leverages changes in harmonic emissions. Arvanitis et al. (140) manually controlled the applied power based on the harmonic and broadband emissions recorded in previous sonications. If an increase in harmonic emissions over baseline was not observed, then the pressure was increased for subsequent sonications. Conversely, a detection of broadband emissions resulted in a decrease in pressure for subsequent sonications. Sun et al. (48) greatly expanded on this and advanced controller development by presenting a fully closed-loop controller based on harmonic emissions that seeks to maintain the level of harmonic emissions within a prescribed range (Figure 3). Recently, the same group has gone further to implement closed-loop control on a clinical system and has tested it in preclinical models (108). Rather than detecting the upper safety bounds, as with sub- and ultraharmonic detection, the initial jump in harmonic emissions is more representative of the lower bound, i.e., the BBB permeability enhancement threshold. Further, beyond the threshold for generating BBB leakage, the enhancement of harmonic emissions over baseline, totaled over the entire treatment, appears to be linearly related to the degree of BBB leakage (48).

Despite the successes of these controllers, sources of variability remain. For example, using inertial cavitation dose, an integration of wideband emissions, Çavuşoğlu et al. (49) recently defined four microbubble behavior bands: the steady oscillation band, the transition zone, the inertial cavitation band, and the high-pressure band. Sudden jumps in inertial cavitation dose delineate the starts of the different bands. They observed that the onsets and the widths of these bands varied substantially between animals, requiring precalibration for each subject. Another source of variability is microbubble dose (85). All of the developed controllers have been tuned for specific microbubble doses, and a change in administered dose will not necessarily be compensated for by the controllers. Thus, variability in administered dose, for example, due to microbubbles breaking during draw-up, mixing, and injection, can impact the degree of BBB permeability enhancement achieved. Receiver characteristics and placement may also impact the recorded signals. While variation in signal strength recorded through bone is a larger source of uncertainty in humans, ultrasound transmission through the rodent skull can still vary substantially (40). The bandwidth and sensitivity of the receiver greatly influence which signals (e.g., subharmonic, harmonic, ultraharmonic, or wideband) can be reliably detected. Furthermore, the dependence on the signal magnitude emitted from a polydisperse microbubble population located in a vascular network may be problematic, since the spatial distribution and microbubble response are largely unknown in clinical scenarios. However, use of the acoustic emissions in some form may ultimately allow the amount of drug delivered to be more precisely prescribed.

While single-element receivers can identify the types of microbubble behavior that are occurring, they are limited in their ability to provide spatial information. A focused receiver will be highly sensitive to cavitation events at its focus, while sacrificing the ability to detect off-target events. Conversely, an unfocused receiver will be sensitive to any events in its field of view but without the ability to separate focal and prefocal cavitation signals; however, it may be possible to determine whether or not emissions are originating in the skull cavity (146).

To achieve spatial mapping of microbubble activity, beyond binary classification, requires an array of receivers in combination with passive beamforming algorithms. Passive beamformers, ranging from simple delay-and-sum approaches (147) to more complex adaptive methods (148, 149), do not rely on absolute time of flight and are thus able to spatially localize cavitation events during long therapeutic ultrasound bursts. A commercial diagnostic probe has been used for passive mapping in nonhuman primates through the temporal bone (150); however, narrow aperture arrays suffer from poor axial resolution using passive beamforming. Superior resolution can be achieved using large aperture hemispherical arrays that take advantage of the skull geometry to achieve tight focusing (50, 151, 152). Going beyond just monitoring the emissions, Jones et al. (50) have integrated active feedback control with 3D spatial mapping during preclinical treatments and have recently demonstrated the ability to predict volumetric bioeffect distributions posttreatment (153). This work relied on a custom device with transmit and receive arrays. As an alternative approach to implementing spatial mapping, Crake et al. (154) designed a sparse receiver array that is compatible with a clinical focused ultrasound brain system.

Monitoring and controlling microbubble activity during BBB permeability modulation is a rapidly developing field, and there is no doubt that the near future will bring new advances. In particular, the increasing availability and affordability of parallel computing approaches, such as general-purpose computing on graphics processing units, will enable the implementation of more complex control algorithms with spatial information in real time.

## CLINICAL TRIALS

Promising results from two decades of preclinical investigations have prompted the first phase of human testing for this drug delivery technique. Thus far, clinical trials have focused on assessing the safety of using ultrasound and microbubble exposures to increase BBB permeability in a variety of pathological contexts, including glioblastoma (23, 155), AD (144, 156), and amyotrophic lateral sclerosis (157). Trials conducted to date have been successful in generating transient increases in vascular permeability without evidence of persistent radiological or neurological abnormalities.

The first in-human demonstration of ultrasound and microbubble-mediated BBB permeability enhancement came in 2014 (23). This trial involved the implantation of an unfocused single-element ultrasound system (SonoCloud-1, CarThera) into the skulls of 17 patients with recurrent glioblastoma. While this approach avoids the complications of transcranial ultrasound propagation, a surgical procedure is required to remove a small portion of the skull and fix the transducer device in place. Using a range of fixed PNPs, researchers repeated sonications two to four times monthly and also administered a chemotherapeutic agent (carboplatin). The authors reported that participants tolerated the procedure well, with no radiological indications of acute hemorrhage, ischemia, or edema. Similarly, no clinical symptoms relating to the procedure were observed in the subsequent hours and days (23). A follow-up report (158) described results from 19 participants with SonoCloud implantation who had received one to ten sonications with carboplatin administration. As reported previously, participants tolerated the procedure well; however, this report described a trend toward efficacy. When comparing median progression-free survival and median overall survival in subjects with no or little sonication-mediated gadolinium contrast enhancement with those with clear enhancement, the latter group displayed trends toward improvement (158).

The first in-human demonstration of transcranial FUS+MB-mediated BBB permeability enhancement came in 2015 as part of a clinical trial conducted at Sunnybrook Research Institute in Toronto, Canada (155). Here, one day prior to surgical resection, peritumoral tissue was targeted in five participants with malignant glioma, in conjunction with chemotherapy administration (liposomal doxorubicin or temozolomide). To correct for skull aberrations, a hemispherical array containing 1,024 elements driven at 220 kHz (ExAblate Neuro, INSIGHTEC) was used with computed tomography-based phase corrections. In each participant, 972–2,430 mm^3^ of tissue was targeted (155). Suitable sonicating pressure amplitudes were determined to be 50% of the input power required to detect subharmonic signals at each targeted volume, on the basis of previous work in a porcine model (159). Gadolinium contrast enhancement was observed in four of five participants following sonication, with no new or worsening neurological signs reported in the 24 h between FUS+MB exposure and tumor resection. Of note, in both cases where sonicated and nonsonicated regions of resected tissue were analyzed, increased chemotherapeutic agent concentrations were found in the targeted tissue (155).

Since this initial work, subsequent trials have sought to test the safety of transcranial FUS+MB exposure in participants with AD (144, 156) and amyotrophic lateral sclerosis (157). These trials were designed to probe for long-term effects and to test the safety of repeated exposures. Thus far, two independent phase I trials have been completed in participants with AD, one targeting the dorsolateral prefrontal cortex (144) and the other targeting the entorhinal cortex/hippocampus (156). Each study reported successful BBB permeability enhancement in the targeted locations with no clinical symptoms believed to be related to the procedure and no persistent vascular leakage evident on CE-T1w images collected 24 h following sonication (i.e., the first follow-up time point). In the Toronto trial, each of the five participants underwent FUS+MB exposure twice, separated by 1 month, with the volume of targeted tissue doubling in the second stage (approximately 350 and 700 mm^3^, respectively). Psychometric tests interrogating cognitive function revealed no clinically significant changes when comparing data collected at baseline to 3 months following the procedures. Acutely, hypointensities in images acquired with a T2*-weighted sequence were observed on the day of sonication in two participants, both of which were resolved within 24 h (144). Additionally, immediately following the procedure, a transient reduction in functional connectivity in the frontoparietal network ipsilateral to sonication was reported, and this reduction recovered to baseline by the next day (160).

In the West Virginia/New York trial (156), each of the six participants underwent FUS+MB exposure three times, separated by 2 weeks, with the volume of targeted tissue ranging from ~350 to 875 mm^3^; the hippocampus and entorhinal cortex were targeted. No radiological signs of overt hemorrhage were observed immediately following sonication, nor were any treatment-related adverse effects or neurological changes observed up to 15 months following the last procedure (156). Several parallel studies in other institutions are ongoing with similar or expanded treatment volumes for AD (ClinicalTrials.gov NCT04118764 and NCT03739905). Given the importance of these targeted brain structures for learning and memory, these studies represent a major milestone in establishing the safety profile of FUS+MB exposure in humans.

To summarize, human trials to date have demonstrated the ability of FUS+MB exposures to generate transient, localized increases in BBB permeability without radiological or clinical indications of persistent tissue damage. Some study participants have reported mild to moderate headaches during the procedure (157) and some have displayed short-lived changes in resting state connectivity (160); however, participants have not exhibited neurological indications believed to be related to FUS+MB exposures in the acute period following the procedure (144, 155, 157). Current trials aim to increase the volume of targeted tissue and the number of procedures each participant undergoes. As the safety profile of this technique is established, future trials will seek to deliver therapeutic agents and assess efficacy.

## FUTURE PERSPECTIVES

In the nearly two decades since the first paper describing reliable BBB permeability enhancement with focused ultrasound and microbubbles (33), there has been tremendous progress in the field. The contributions from many teams worldwide working in this arena have not only improved our understanding of the effects of ultrasound and microbubbles on the brain but also have facilitated the translation to clinical investigations. We understand now that the induced bioeffects go far beyond enhanced permeability to enable therapeutic agent delivery, and, despite the large volume of work done to date, the future will no doubt bring even more insight into cellular mechanisms and effects that may be leveraged in novel therapeutic approaches. Where possible, efforts should be directed at developing strategies that take advantage of both drug delivery potential and the induction of bioeffects to synergistically produce more efficacious outcomes. To this end, we must work to further characterize the time course of events that follow various regimes of BBB permeability enhancement, as research in the field continues to reiterate the idea that the initial impact on cerebrovascular leakage greatly influences downstream tissue effects. Equally clear is the fact that controlled exposures that can select for specific bioeffects are key to maximizing the potential of this technology and achieving widespread adoption. Further, means of decoupling treatments from MRI guidance, such as through the use of neuronavigation (161), devices with acoustic registration (154, 162) and monitoring (50), or the development of lower-cost devices (29, 163), will improve the affordability of these procedures. To see this technology reach the first stages of clinical investigation has been truly exciting. Going forward, it will be critical for the field to identify key treatment indications for larger multicenter trials to prove the effectiveness of this intervention. Although there are many questions that remain to be answered, the present state of the field holds great promise, and the future is bright.
